# Role of Antibiotic Cement Coated Nailing in Infected Nonunion of Tibia

**DOI:** 10.5704/MOJ.1703.019

**Published:** 2017-03

**Authors:** C Bhatia, AK Tiwari, SB Sharma, S Thalanki, A Rai

**Affiliations:** Department of Orthopaedics, Government Medical College, Kota, India

**Keywords:** nonunion, infected, antibiotic, cement, nailing

## Abstract

**Introduction:**

Infected nonunion of long bones is a chronic and debilitating disorder. It is more difficult to deal with when the implant used for internal fixation itself becomes a potential media for infection because of bacterial adhesion and biofilm formation. Traditionally, it is managed by two-stage procedure for controlling the infection first and then treating the nonunion. This study has been undertaken to explore antibiotic cement coated nailing as single stage treatment modality for treating infection and achieving stability at the same time.

**Materials and Methods:**

Twenty patients (above 18 years of age) with infected nonunion of tibia with bone gap less than 2 cm were managed using antibiotic cement coated K-nail. Antibiotic cement nail was prepared using endotracheal tube method. Antibiotics used were a combination of vancomycin and teicoplanin.

**Results:**

Infection was controlled in 95% of the patients. Bony union was achieved in 12 of 20 (60%) patients with antibiotic cement nailing as the only procedure with average time of union of 32 weeks. Remaining 8 patients required additional procedures like bone grafting or exchange nailing and these were done in six patients, with union of fracture. Two patients refused to undergo further procedures. Complications encountered were difficult nail removal in three cases, broken nail in two cases, and bent nail in one case. Recurrence of infection was observed in two patients. Average period of follow-up was 13 months.

**Conclusion:**

Antibiotic cement impregnated nailing is a simple, economical and effective single stage procedure for the management of infected nonunion of tibia. It is advantageous over external fixators, as it eliminates the complications of external fixators and has good patient compliance. The method utilizes existing easily available instrumentation and materials and is technically less demanding, and therefore can be performed at any general orthopaedic center.

## Introduction

Infected nonunion of long bones is a chronic and debilitating disorder that still poses a very complex problem to the surgeon today in terms of cost and time-effective treatment^1^. Various factors contribute to infected non-unions, including open fractures, loss of soft tissue or bone, infection after internal fixation, chronic osteomyelitis with pathologic fractures, and surgical debridement of infected bone^2^. It is more difficult to deal with when the implant used for internal fixation itself becomes a potential media for infection because of bacterial adhesion and biofilm formation^3^.

Most of the orthopaedic trauma infections are caused by biofilm-forming bacteria ^3^. Biofilm consists of hydrated matrix of polysaccharide and protein. Once formed, it protects the microorganism from antimicrobials, opsonization, and phagocytosis, thus contributing to the chronicity of infections^4^. In order to cure biofilm-related infection, four principles formulated by Cierny and Mader must be observed: (1) complete surgical debridement with dead space management, (2) fracture/nonunion stabilization, (3) soft tissue coverage, and (4) adequate antibiotic levels^5^.

Traditionally, treatment of an infected nonunion follows a two-stage procedure. The first stage comprises of debridement with or without antibiotic cement bead insertion and systemic antibiotics to convert an infected nonunion to an aseptic nonunion. The second stage is performed to achieve stability by either external or internal fixation with or without bone grafting^2,6-10^.

The use of an antibiotic impregnated cement coated IM nailing for infected nonunion of tibia and femur fractures has been well-documented in the literature^11-18^. The cement nail provides stability across the fracture site, unlike cement beads and osseous stability is important in the management of an infected nonunion ^17,18^. Secondly, antibiotic cement allows higher concentration of antibiotic at the local site than is achievable with systemic antibiotics and is associated with fewer side effects. Antibiotic cement has been shown to elute antibiotic at the local sites for up to 36 weeks thus having a therapeutic effect on refractory infection^16,17^. Hence, unlike traditional methods of management of infected nonunion antibiotic cement coated nailing acts as a single stage procedure by providing stability and treating infection at the same time along with other advantages like early mobilization, avoidance of pin site infections, ease of performance and being cost effective.

Antibiotics used for this purpose should have special properties such as broad spectrum of activity, heat stability, and low allergenicity. Gentamicin has been the most widely used agent followed by vancomycin^19,20^. In our study, we used the combination of vancomycin with teicoplanin as both have the desired properties. The purpose of our study was to evaluate the outcome of antibiotic cement coated nailing in the management of infected nonunion of tibia in terms of infection control and bony union.

## Materials and Methods

This is a prospective study of 20 patients (19 males, one female) with infected nonunion of tibia aged from 22 to 61 years (mean, 39 years) who were treated using antibiotic cement coated nailing. Patients with diaphyseal fractures of tibia with bone gap less than 2 cm were included in the study. Patients who were allergic to vancomycin or teicoplanin were excluded. Of these 20 patients, thirteen sustained open fractures, and seventeen had undergone one or multiple procedures. Twelve patients had positive preoperative cultures for staphylococcus aureus of which ten were resistant to gentamicin. Eight patients had cultures that were negative despite clinical evidence of infection.

After thorough preoperative evaluation, informed consent were taken for surgery. The first step in cases previously operated involved removal of the implant. This was followed by thorough debridement of the infected bone and soft tissues with copious lavage. Specimens of the bone, soft tissues and any purulent material were sent for culture and sensitivity. After that, intramedullary canal was adequately reamed and prepared to fit a larger diameter nail, it was thoroughly washed with saline.

The surgical team then changed their gowns and gloves. The limb was prepared again and re-draped. An appropriate size antibiotic impregnated nail was prepared on a separate sterile table. The required length of the nail was determined with the same standard method used to determine the length of interlocking nail. Kuntscher nail (K-nail) of 6 or 7 mm diameter was chosen and coated with bone cement up to 1 mm less than the diameter of the last reamer used. 40 gm of cement was thoroughly mixed with 2 gm vancomycin and 2 gm teicoplanin, following which the polymer was added. An endotracheal tube of the internal diameter same as the desired diameter of the cement coated nail was then filled with the doughy mixture of antibiotics and cement. K- nail of diameter 2 mm less was then pushed through this endotracheal tube and allowed to set for 10-15 minutes. The endotracheal tube was then cut open using a surgical knife to retrieve the K- nail uniformly coated with antibiotic cement. Eyes of the nail were kept uncovered to facilitate easy removal later. The nail was then inserted antegrade in the tibia. Nail-cement debonding during insertion was avoided by allowing adequate time for the cement to set and bond with the nail.

Post-operatively, the wound was inspected at 48-72 hours intervals and the patient was administered intravenous antibiotics as per culture and sensitivity reports for 2-4 weeks. The patient would then be discharged on oral antibiotics for a time period depending on individual patient characteristic, condition of the wound and the organism involved.

As soon as the wound healed, a patellar tendon-bearing cast or brace was applied and gradual weight bearing was permitted. The cast was changed every six weeks and continued till union was confirmed based on clinical and radiological assessments. Active physiotherapy for regaining ankle and knee mobility was instituted till the range of movement was satisfactory. Patients were followed up every week for first one month after discharge, then once a month for three months and then once every 2-3 months till the final follow-up. The average period of follow-up was 13 months.

Patients were evaluated in terms of infection control and bony union. They were divided into following categories (1) infection controlled, sound bony union (2) infection controlled, signs of fracture healing with partial union (3) infection controlled, no signs of fracture healing (4) infection persisting, no signs of fracture healing. Depending on the above, it was decided which patients needed further intervention like bone grafting or exchange nailing, etc.

## Results

After an average follow-up of 13 months, infection was controlled in 19 of 20 (95%) patients (Table I). Bone union was achieved in 18 of 20 patients with or without additional procedures (Table II). Eight patients required additional procedures in order to treat infection or achieve bone union. Six of the eight patients achieved union after additional procedures (Table III). One patient with persisting infection and one patient with controlled infection refused to undergo any further procedures. One patient who presented with bent antibiotic cement nail in situ with infection still persisting underwent exchange nailing with a new antibiotic cement nail and went on to achieve bone union with no additional procedure. Hence, in 12 of 20 patients (60%), antibiotic cement nailing was the only procedure required to achieve infection control and bone union.

**Table I:** Control of infection

	No. of cases	Percentage
Infection controlled	19	95%
Infection not controlled	1	5%

**Table II:** Bony union

	No. of cases	Percentage
Union without additional procedure	12	60%
Union with additional procedure	6	30%
No Union	2	10%

**Table III:** Additional procedures

Procedure	No. of patients
Bone grafting	3
Exchange inter-locking nail with bone grafting	3
Refused	2

**Fig. 1:**
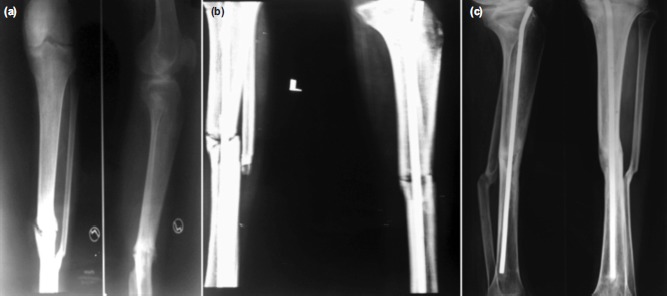
(a) Pre-operative radiograph of a patient with infected nonunion of tibia, (b) Postoperative radiograph after debridement and antibiotic cement coated nailing and (c) Radiograph showing bony union achieved at 28 weeks.

Average time taken for union was 38 weeks, minimum being 24 weeks and the maximum was 44 weeks. Average time taken for union in patients in whom antibiotic cement nailing was the only procedure required was about 32 weeks. (Fig. 1a,b,c)

**Fig. 2:**
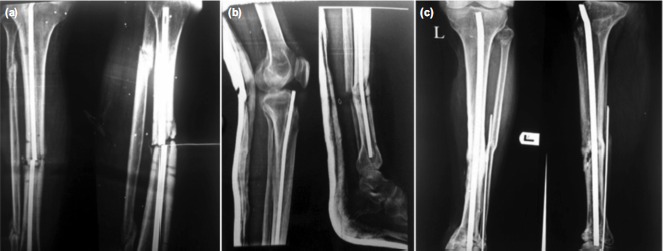
(a) Broken nail, (b) Bent nail and (c) Nail migration into the ankle joint.

The complications observed in our study were: difficult nail removal in three patients, nail breakage in two patients (Fig. 2a), nail bending in one patient (Fig. 2b) and nail migrating into the ankle joint (Fig. 2c) in one patient. Two cases also presented with recurrence of infection.

## Discussion

The treatment of infected nonunion requires procedures to control the infection and to provide stability in order to achieve union ^14^. There is no single universally accepted modality of treatment presently available for the management of infected nonunion. Traditionally, infected nonunions have been managed using two-step procedure to control the infection first and subsequently to treat the nonunion.

Delivery of antibiotics to the infection site systemically or locally is essential in order to control infection. Long term infection and repeated debridement create excessive fibrosis around the nonunion site and hinder antibiotic permeability^21^. Hence, delivery of antibiotics to the local site is far more beneficial than systemic administration of antibiotics. The use of antibiotic-impregnated polymethylmethacrylate cement beads for local delivery of antibiotics without any systemic toxicity has been well documented for the management of osteomyelitis and open fractures ^22,23^. However, these antibiotic cement beads do not provide any stability across fracture site and cannot be placed in the intramedullary canal as it entails difficult removal due to fibrous ingrowths.

The antibiotics that are used for this purpose should have a broad spectrum of activity, should be heat stable, have good elution properties from the cement and should have low allergenicity. Most of the researchers in past used a combination of vancomycin with gentamicin or tobramycin ^11,14,19,30,31^. In our study, we used a combination of vancomycin and teicoplanin as most of the culture and sensitivity reports from the patients at our center were reporting staphylococcus aureus which was resistant to gentamicin. There have been reports of changing patterns of resistance in microorganisms, partly as a result of widespread use of antibiotics for local and systemic prophylaxis. Increasing numbers of gentamicin-resistant species are being reported to cause deep infections, including medullary infections ^24,25^. Our study is unique in this aspect as no other researcher in the past have used teicoplanin, although stability and biocompatibility of this antibiotic in bone cement is well established ^26,27^. The concomitant use of two antibiotics also helps widen the spectrum of activity.

Stability across fracture site can be achieved by external or internal fixation. However, a high prevalence of pin-site infections, muscle contractures, and joint stiffness has been observed in association with external fixation ^27,28^. Ilizarov fixator, which is an excellent modality to treat infected nonunion, is a cumbersome assembly and is technically demanding and also has a low acceptability among patients. When internally fixed the implants used for internal fixation act as foreign body and can be a potential media for infection with the formation of biofilms. The presence of foreign body and biofilm makes the eradication of infection more difficult by systemic antibiotics.

Antibiotic cement impregnated nails deliver a high concentration of antibiotics at the local site without causing any systemic toxicity along with providing stability at the nonunion site, thereby converting a two stage procedure of treating nonunion into a single stage procedure. Antibiotic cement nails also help avoid complications like pin site infections, joint stiffness, muscle contractures as the patient can be mobilized early. In following the initial report by Paley and Herzenberg in 2002 ^11^, many researchers have produced good results using this procedure for the treatment of infected nonunion. Paley and Herzenberg studied a small sample of nine cases and reported infection control in all the cases. Thonse and Conway in 2007 published a study with a large sample size of 52 patients and reported infection control in 85% of the patients ^14^. Qiang *et al* reported infection controlled in 17 of 18 (94.4%) cases^12^. In our study, infection was completely eradicated in 95% of the cases. Similar results suggest that antibiotic cement impregnated nailing is definitely a good means to eradicate infection.

In terms of bony union, the results vary in different studies. Thonse and Conway reported bony union in 73% of the cases with antibiotic nail as the index procedure. Qiang *et al *reported it as index procedure in about 22 % cases, the same being 60 % in our study. Thonse and Conway had used interlock nails for the preparation of antibiotic cement rods. Sancheti *et al*^30^ published a study in which 22 of 25 patients required a secondary procedure. They had divided the patients into three groups according to the bone gap and reported that infection control and union rate were both poor in patients with increasing bone gap.

In our study, two patients reported with broken nail and one patient with bent nail which was due to non-compliance and early unprotected weight bearing. Another complication observed in our study was difficult nail removal, seen in three patients, which may be attributed to improper nail preparation and late presentation to hospital for removal. The nail may get incarcerated inside the medullary canal due to fibrous growths and cement nail bonding. Recurrence of infections occurred in two patients in our study. It was also reported by a few other researchers like Qiang *et al*^12^, Selhi *et al*^31^. Timely removal of nail is recommended once the infection has subsided because theoretically live bacteria are known to persist on antibiotic impregnated cement under in vitro conditions ^32^.

## Conclusion

Antibiotic cement impregnated nailing is a simple, economical and effective single stage procedure for the management of infected nonunion of tibia. It is advantageous over external fixators, as it eliminates the complications of external fixators and has good patient compliance. The method utilizes existing easily available instrumentation and materials and is technically less demanding, and therefore can be performed at any general orthopaedic center.
